# COVID-19 Antibody Response in Patients with Thalassemia

**DOI:** 10.7759/cureus.40567

**Published:** 2023-06-17

**Authors:** Nidhi Kumari, Sunil Gomber, Pooja Dewan, Shiva Narang, Rafat Ahmed

**Affiliations:** 1 Pediatrics, University College of Medical Sciences, Delhi, IND; 2 Medicine, University College of Medical Sciences, Delhi, IND; 3 Biochemistry, University College of Medical Sciences, Delhi, IND

**Keywords:** igg antibodies, sars-cov-2, covid-19, india, covid-19 in thalassemia, sars-cov-2 antibody response, thalassemia

## Abstract

Background

The coronavirus disease 2019 (COVID-19) can severely affect people with comorbidities such as those with diabetes, hypertension, chronic lung disease, cancer, and hemoglobinopathies. Studies assessing the clinical characteristics and immune response to COVID-19 infection in patients with thalassemia are limited.

Objectives

The primary objective of the study was to study the clinical pattern and the immunoglobulin G (IgG) antibody response to severe acute respiratory syndrome coronavirus 2 (SARS-CoV-2) in patients with transfusion-dependent thalassemia (TDT) compared to patients without thalassemia. The secondary objective wasto study the relationship of COVID-19 severity with IgG antibody titers.

Setting, Design, and Participants

This case-control study was conducted at a tertiary care hospital between January 2021 and August 2022. A total of 30 patients with TDT (mean age: 12.7 years, SD: 4.7) and 30 patients without thalassemia (mean age: 13.9 years, SD: 7) who tested positive for COVID-19 in the preceding six weeks were recruited.

Methods

Serum samples from the cases and controls were collected after 6, 12, and 24 weeks of COVID-19 infection for IgG antibody estimation using chemiluminescent immunoassay.

Outcome variables

The primary variable was comparative analysis of antibody levels and clinical profile of COVID-19 in cases and controls. The secondaryvariable was association of the severity of COVID-19 with the antibody titers produced.

Results

Symptomatic individuals among cases (n=12) were significantly lesser than controls (n=22) (p=0.009). The median IgG titers of cases and controls were comparable at six weeks (p=0.40), but the titers were significantly lower for cases at 12 weeks (p=0.011) and 24 weeks (p=0.006). There was significant fall in titers from 6 to 12 and 24 weeks in both the groups. The titers were not affected by COVID-19 severity and pre-existing comorbidities.

Conclusion

Patients with TDT manifest with mild or asymptomatic COVID-19 and mount a comparable IgG antibody response to COVID-19 akin to controls. However, this serological response could not sustain over three to six months advocating the need for protection through vaccination.

## Introduction

The coronavirus disease 2019 (COVID-19) caused by the severe acute respiratory syndrome coronavirus 2 (SARS-CoV-2) has led to extensive morbidity and mortality worldwide. Mutations in the viral structure made it extremely difficult to treat and synthesize effective vaccines against the disease. It took nearly six months for China to declare the first COVID-19 vaccine for limited use in military in June 2020. Since then, multiple vaccines have now been manufactured by different nations [1], and a large population across the globe has been vaccinated against SARS-CoV-2. However, the long-term effects of the disease and vaccinations are yet to be discovered.

Host factors significantly determine the course of illness, its progression, and the outcome of COVID-19. Individuals with pre-existing comorbidities such as diabetes, hypertension, cancer, and hemoglobinopathies are at high risk of severe infection and worse outcomes [2]. Hemoglobinopathies such as thalassemia and sickle cell disease are common monogenic disorders that are associated with multi-system involvement and require life-long therapy and follow-up [3-5]. Their disease course is often complicated by multiple sequalae, particularly in developing countries where the infrastructure to manage such diseases is lacking. Hence, these patients are at an increased risk of severe complications in the COVID-19 pandemic [6-7].

Beta-thalassemia has been associated with a wide spectrum of immunological defects [8-9]. Early studies [10-11] showed no significant differences in the serum immunoglobulin assays of thalassemia patients and non-thalassemia controls. However, it is believed that frequent blood transfusions over years and chronic iron overload lead to significant blunting of the lymphoproliferative responses in thalassemia patients [8,11-13]. Impaired B-cell differentiation has also been noted [9]. Thus, thalassemia patients have been discerned to be immunocompromised [14] and can be considered as a vulnerable patient population to the complications of a comorbid condition such as COVID-19. Hence, this study was aimed at evaluating clinical and immunological response of patients with thalassemia to COVID-19 infection in comparison with controls.

## Materials and methods

This case-control study was conducted in the Departments of Pediatrics, Medicine, and Biochemistry of a tertiary care hospital between January 2021 and August 2022. Thirty TDT patients were recruited from the thalassemia day care center who had tested positive for COVID-19 in the preceding six weeks by reverse transcription polymerase chain reaction (RT-PCR)/rapid antigen detection/Truenat®. Thirty age- and sex-matched controls without thalassemia who had tested positive for COVID-19 in the preceding six weeks were also recruited from the outpatient department. Appropriate informed consent was taken. Patients on immunosuppressive therapies and extremely malnourished for age or with underlying chronic liver/kidney disease were excluded. Institutional ethics committee approval was taken.

Baseline data

Baseline characteristics including demographic profile, anthropometry, relevant past history, COVID-19 symptomatology, and treatment history of all participants were noted. Additional data regarding splenectomy status, pre-COVID serum ferritin level, iron overload in the heart and liver (as measured by T2*MRI), blood group, and pre-existing comorbidities such as hypothyroidism, hypogonadism, and diabetes in the thalassemia patients were obtained from their records.

SARS-CoV-2 IgG assay

The SARS-CoV-2 immunoglobulin G (IgG) antibodies were estimated using Beckman Coulter’s Access 2 SARS-CoV-2 IgG assay machine (Beckman Coulter, Brea, CA). A total of 2 mL of whole blood was collected in plain vials from each candidate at 6, 12, and 24 weeks after COVID-19 infection. A total of 20 µL of freshly separated serum was added to a reaction tube with buffer, and paramagnetic particles were coated with recombinant SARS-CoV-2 protein, which is specific for the receptor-binding domain of the S1 protein. After incubation in this vessel, particles that were attached to the solid phase were held in a magnetic field, and those unattached were washed away. Next, a monoclonal anti-human IgG alkaline phosphatase conjugate was added, which binds to the IgG antibodies captured on these particles. A wash step removed the unbound conjugate. A chemiluminescent material was then added to the vessel, and the light generated by the reaction was quantified with a luminometer. This light production was compared to the cut-off value of the machine. A reactive titer was defined as ≥1.00 S/CO (signal-to-cut-off ratio).

Outcome variables

IgG antibody levels and clinical profile of COVID-19 in patients with thalassemia were assessed in comparison to controls (primary outcome variable). The association of COVID-19 severity with the IgG antibody titers was evaluated (secondary outcome variable).

Statistical analysis

The data were summarized and analyzed using the statistical software SPSS Version 25 (IBM Corp., Armonk, NY). The continuous baseline variables were compared using unpaired t-test, and categorical variables were compared with the chi-square test. The Friedman test was used for within-group titer comparisons (6, 12, and 24 weeks), followed by the Wilcoxon signed rank test with Bonferroni adjustment. The Mann-Whitney U test was used for comparison between cases and controls at different time points of study (6, 12, and 24 weeks). The Chi-square test was used to compare the reactivity of asymptomatic and mildly symptomatic patients among cases and controls at all three time points. A p-value of <0.05 was considered significant.

## Results

**Figure 1:**
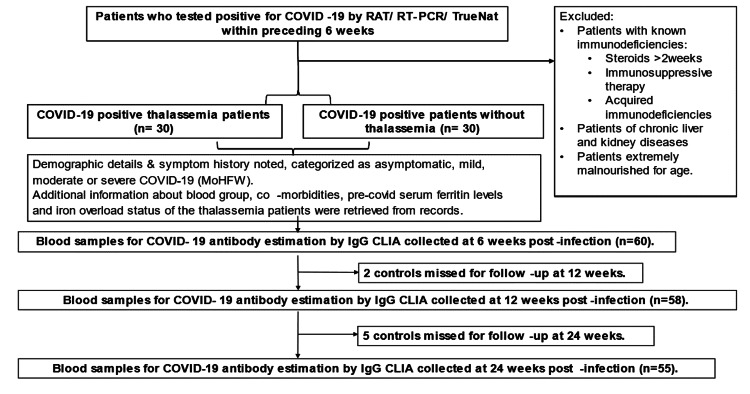
Flow diagram of the study

Between January 2021 and August 2022, 30 TDT patients (20 males, 10 females) (mean age: 12.7 years, SD: 4.7) who tested positive for COVID-19 were recruited as cases; 30 individuals (13 males, 17 females) without thalassemia (mean age: 13.9 years, SD: 7) who tested positive were enrolled as controls. The age (p=0.45) and gender (p=0.52) compositions of both groups were statistically comparable. These participants were followed for a period of 24 weeks (Figure 1).

Clinical characteristics

**Figure 2:**
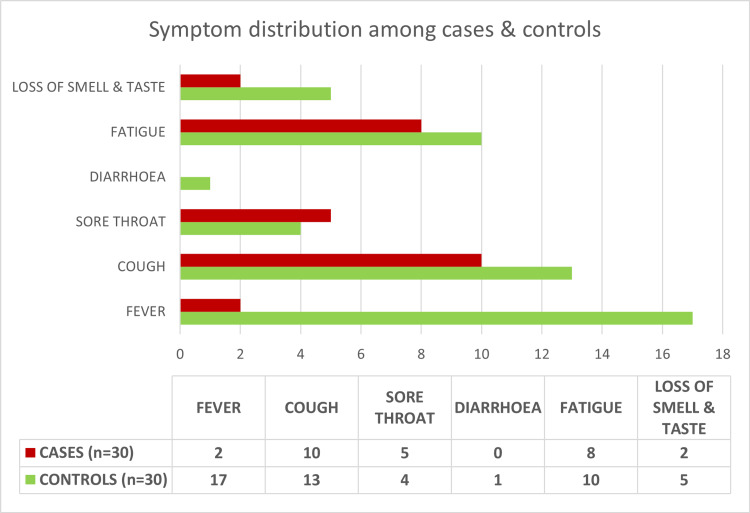
Frequency distribution of symptoms among both groups

In this study, 56.6% (34/60) patients were mildly symptomatic as defined by the Ministry of Health and Family Welfare, India [15-16], and rest were asymptomatic with no COVID-19-related symptoms. The number of symptomatic individuals among cases (12/30) was significantly lower than controls (22/30) (p=0.009). Cough was the most common symptom among cases (33%), while fever was the most common among controls (57%) (Figure 2). The mean (SD) duration of illness was 5 (1.6) days in the symptomatic cases and 4 (1.5) days in the symptomatic controls. The difference was statistically insignificant (p=0.07).

SARS-CoV-2 IgG titers

Six Weeks

Titers were measured for 60 patients (30 cases, 30 controls). Among cases, 13 out of the 18 asymptomatic patients, and 11 out of the 12 mildly symptomatic patients had reactive antibody titers (≥1.0 S/CO). The median titer for the 30 cases was 4.36 S/CO (IQR: 1.32-11.65).

Among controls, all eight asymptomatic patients and 19 out of the 22 mildly symptomatic patients had reactive antibody titers. The median titer for the 30 controls was 7.04 S/CO (IQR: 2.35-13.09).

The number of patients having reactive titers was statistically similar in asymptomatic and mildly symptomatic patients across both groups (p-value for cases=0.35; p-value for controls=0.54) (Table 1). Also, the difference in the median titers across the two groups was not statistically significant (p=0.40).

**Table 1:** Comparison of reactivity among asymptomatic and mild patients *Fisher's exact test applied

	Asymptomatic		Mild		
	Reactive	Non-reactive	Reactive	Non-reactive	p-Value
6 weeks					
Cases	13	5	11	1	0.35*
Controls	8	0	19	3	0.54*
12 weeks					
Cases	10	8	7	5	0.88
Controls	7	0	16	5	0.29*
24 weeks					
Cases	1	17	3	9	0.27*
Controls	4	4	6	11	0.66*

Twelve Weeks

Titers were measured for 58 patients (30 cases, 28 controls). Among cases, 10 out of the 18 asymptomatic patients and seven out of the 12 mildly symptomatic patients had reactive antibody titers. The median titer for cases was 1.19 S/CO (IQR: 0.41-3.26).

Among controls, all seven asymptomatic patients and 16 out of the 21 mildly symptomatic patients had reactive antibody titers. The median titer for the 28 controls was 3.23 S/CO (IQR: 1.55-7.14).

The number of patients having reactive titers was statistically similar in asymptomatic and mildly symptomatic patients across both groups (p-value for cases=0.88; p-value for controls=0.29). However, the difference in the medians across the two groups was statistically significant (p=0.01).

Twenty-Four Weeks

Titers were measured for 55 patients (30 cases, 25 controls). Among cases, one out of the 18 asymptomatic patients and three out of the 12 mildly symptomatic patients had reactive antibody titers. The median titer for cases was 0.51 S/CO (IQR: 0.18-0.88).

Among controls, four out of the eight asymptomatic patients and six out of the 17 mildly symptomatic patients had reactive antibody titers. The median titer for controls was 0.84 S/CO (IQR: 0.55-3.19).

The number of patients having reactive titers was statistically similar in asymptomatic and mildly symptomatic patients across both groups (p-value for cases=0.27; p-value for controls=0.66). However, the difference in the medians across the two groups was statistically significant (p=0.006).

Trend of Titers

The fall in titers from 6 weeks to 12 weeks and then to 24 weeks in both cases and controls was statistically significant (p<0.05).

The mean (SD) pre-COVID serum ferritin level in the cases was 2,670 (1,318) ng/Ml. Various comorbidities were noted in these patients (Table 2). No significant correlation was noted between these conditions and severity of disease or antibody titers (p>0.05). No comorbidities were noted in the control group.

**Table 2:** Comorbidities in patients with thalassemia (obtained from records)

Comorbidity	No. of patients (percentage of total)
Hypothyroidism	3 (10%)
Hypogonadotropic hypogonadism	4 (13.3%)
Splenectomized	2 (6.6%)
Hepatic iron overload (T2*MRI)	
Mild	2 (8.7%)
Moderate	5 (21.7%)
Severe	12 (52.2%)
Cardiac iron overload (T2*MRI)	
Mild	1 (4.3%)
Moderate	1 (4.3%)
Severe	5 (21.7%)

Blood groups of patients with thalassemia were obtained from records. Ten patients were B+ (34%), eight patients were O+ (27%), five patients were AB+ (17%), four patients were A+ (13%), and one patient each was O-, AB-, and B-. No significant correlation of blood group was noted with antibody titers (p>0.05).

## Discussion

The studies conducted on thalassemia patients with COVID-19 are sparse [17-21] (Table 3), and to the best of our knowledge, this is a pioneer case-control study to assess the IgG antibody response of TDT patients post-COVID-19 infection.

**Table 3:** Previous studies of COVID-19 infection in patients with thalassemia

Author, Year	Place of study	Age group	Sample size	Outcome
Karimi et al., 2020 [20]	Iran	35.5 ± 11.5 years	43 (11 NTDT, 32 TDT)	Prevalence of COVID-19 was significantly higher in NTDT patients than the general population. Mortality due to COVID-19 was significantly higher in thalassemia patients than the general population.
Motta et al., 2020 [18]	Italy	44 ± 11 years	11 (10 TDT, 1 NTDT)	No increase in severity or mortality in thalassemia patients as compared to the general population affected by SARS-CoV-2.
Telfer et al., 2020 [19]	UK	33 years (6 weeks to 92 years)	26 (20 TDT, 6 NTDT)	Favorable prognosis apart from two deaths, which had their independent risk factors.
Jean-mignard et al., 2020 [21]	France	11-60 years	16 (15 TDT, 1 NTDT)	High rate of hospitalization for a younger age. Favorable outcome attributed to low incidence of severe hemochromatosis.
Bou-Fakhredin et al., 2021 [17]	Lebanon	30.7 years (9–61)	40 (27 TDT, 13 NTDT)	No deaths reported, no correlation between blood group and clinical course of disease.

In the present study, 60% (18/30) of the patients with thalassemia and COVID-19 were asymptomatic akin to the Lebanese study by Bou-Fakhredin et al. [17]. However, unlike their study, all the symptomatic cases in our study population were mild, and none required oxygen support or hospitalization. They recovered with symptomatic management. The number of symptomatic patients was significantly higher (p=0.009) in the control group (22/30), but all remained mild in severity. The lesser severity of COVID-19 could be related to the younger age composition of this study (14 months to 28 years) as younger age has been hypothesized as a protective factor for severe COVID-19 [22]. A meta-analysis by Zimmermann and Curtis [23] has postulated reasons for milder infections in children, which include differences in innate and adaptive immunity, more frequent recurrent and concurrent infections, pre-existing immunity to coronaviruses, differences in microbiota, higher levels of melatonin, protective off-target effects of live vaccines, and lower intensity of exposure to SARS-CoV-2.

Among cases, 21 (70%) out of 30 had at least one comorbidity. Moderate-to-severe hepatic iron overload was noted in 57% (17 of 30) of the thalassemia patients, whereas 20% (6 of 30) had moderate-to-severe cardiac iron overload. However, appropriate chelation was provided, and 100% of these patients had normal liver function tests and two-dimensional echocardiography. Three patients had underlying hypothyroidism (on levothyroxine), four had hypogonadotropic hypogonadism (all males on monthly testosterone), and two were splenectomised (taking penicillin). None of these patients experienced an increased severity of symptoms, and there was no significant correlation between these comorbidities and the antibody titers. These findings reiterate the fact that adequate management of iron overload-related conditions in thalassemia can prevent severe morbidity and mortality due to COVID-19 [18,19]. There were no comorbidities noted in the control group.

The present study stands in sharp contrast with the Iranian study by Karimi et al. [20], which reported an increased rate of mortality due to SARS-CoV-2 in β-thalassemia (18.6%) as compared to the general population in Iran (4.7%) (p<0.001). However, their study was limited by a small sample size of COVID-19-positive thalassemia patients, which could have been inadequate to make generalized comparisons with a country’s population.

We did not find any significant correlation between blood group and risk or severity of COVID-19, which stands in contrast to most previous studies [24-26] except the Lebanese study [17], which showed no statistical correlation between the blood groups and the clinical course of disease (p=0.63). This difference may have been due to the small sample size of this study or because of some racial or demographic features.

This study is the first to evaluate the IgG antibody response to SARS-CoV-2 infection in patients with thalassemia. It was found that patients with thalassemia mount a statistically comparable antibody response to COVID-19 measured at six weeks post-infection. However, when the titers were subsequently measured at 12 weeks (30 cases, 28 controls) and 24 weeks (30 cases, 25 controls), they were significantly lower in the cases (p-value of 0.011 at 12 weeks; p-value of 0.006 at 24 weeks). There was statistically significant fall of titers from 6 to 12 to 24 weeks in both the groups, but the fall was more in the cases. This suggests that thalassemia patients mount comparable IgG antibody response to SARS-CoV-2 at the outset, but they have a rapid decline of antibodies over initial months of infection, more than those without thalassemia. However, studies with larger sample size are needed to confirm these findings.

It was noted that even asymptomatic and mildly symptomatic patients, with or without thalassemia, could generate substantial IgG antibodies. However, as we did not have any patients with moderate or severe disease, comparison of titers between asymptomatic/mild and moderate/severe COVID-19 patients could not be done.

Five cases and two controls did not have reactive titers (≥1.0 S/CO) at any time point, which could mean that these patients either never mounted adequate serological response or had a rapid decline in the IgG antibodies by six weeks of convalescence. It has been previously noted that around 5% of RT-PCR positive patients may have negative antibody titers [27].

None of our patients reported re-infection with COVID-19 during the six months of follow-up, similar to the findings of Lumley et al. [28]. Also, four out of 30 cases (13%) and 10 out of 25 controls (40%) had reactive IgG antibodies at 24 weeks. This could mean that antibodies to SARS-CoV-2 may persist beyond 6 months post-infection in some patients as shown in multiple studies [29-30].

## Conclusions

The present study concludes that most patients with transfusion-dependent thalassemia manifest with mild or asymptomatic COVID-19. Also, these patients are able to mount a statistically comparable IgG antibody response to COVID-19 akin to controls. However, the serological response cannot sustain over three to six months as they have a more rapid fall in antibody titers when compared to the control group. This emphasizes the need for protection of this vulnerable group by immunization.
